# Acute influence of resistance exercise on basketball shooting mechanics and accuracy

**DOI:** 10.3389/fspor.2023.1272478

**Published:** 2023-10-25

**Authors:** Dimitrije Cabarkapa, Damjana V. Cabarkapa, Anthony B. Ciccone, Shay M. Whiting, Nicolas M. Philipp, Drake A. Eserhaut, Andrew C. Fry

**Affiliations:** ^1^ Jayhawk Athletic Performance Laboratory—Wu Tsai Human Performance Alliance, Department of Health, Sport and Exercise Sciences, University of Kansas, Lawrence, KS, United States; ^2^ Department of Exercise Science and Outdoor Recreation, Utah Valley University, Orem, UT, United States

**Keywords:** biomechanics, performance, training, sport, coaching, free-throw, jump-shot

## Abstract

The purpose of the present study was to examine the acute impact of resistance exercise on basketball shooting mechanics and accuracy. Ten resistance-trained recreationally active men with previous basketball playing experience (x̄ ± SD; height = 182.6 ± 9.7 cm; body mass = 79.2 ± 13.9 kg; age = 25.6 ± 5.5 years) performed control, upper-body, and lower-body training sessions in randomized order followed by 5 sets of stationary free-throw (4.57 m), two-point (5.18 m) and three-point (6.75 m) basketball shooting drills in 30 min time increments. Each testing session was separated 3–7 days apart. Kinematic variables during both the preparatory and release phases of the shooting motion were derived from a high-definition camera recording at 120 fps positioned 10 m away perpendicular to the participant's shooting plane of motion. Restricted maximum likelihood linear mixed-effects model analysis revealed that a combination of all fixed effects could account for <1% of the total variance in each dependent variable pertaining to basketball shooting mechanics. A 9.9–11.8% decrease in two-point and three-point shooting accuracy was observed immediately following an upper-body training session. However, the observed performance suppression disappeared 30 min post-exercise completion. Overall, the findings suggest that performing upper-body or lower-body resistance training prior to on-court practice sessions has no impact on free-throw, two-point, and three-point biomechanical parameters examined in the present study and a minor acute impact on mid-range and long-range shooting accuracy in male basketball players.

## 1. Introduction

Basketball is one of the most popular international sports. It is a fast-paced game that requires players to possess well-developed physical performance characteristics in order to properly respond to on-court competitive demands (1). Some of these characteristics include strength, power, speed, agility, as well as anaerobic and aerobic capacity (1–4). Collectively, they all play an integral role in achieving peak performance on various basketball-specific tasks in order to secure the winning game outcome such as rebounding, shooting, and sprinting (5–8).

Based on the currently available scientific literature, maximal strength seems to be the most prominent among the previously mentioned physical performance attributes. A considerable amount of research reports have documented a positive relationship between upper-body and lower-body strength and basketball-specific performance (1–3, 8–11). For example, Dawes et al. (3) found a strong positive relationship between playing time and bench press (*r* ≥ 0.71) and back squat (*r* ≥ 0.74) one-repetition maximum (1RM) within a cohort of National Collegiate Athletic Association (NCAA) Division-II male basketball players. Similar findings pertaining to the positive relationship between playing time and back squat 1RM (*r* = 0.52–0.64) were observed by Hoffman et al. (9) when examining athletes competing at the NCAA Division-I level. Lower-body maximal strength has been shown to be a physical performance characteristic with the highest correlation with playing time (9). Yet, it should be noted that the aforementioned association was nonexistent (*r* = 0.16) prior to the start of the strength training program. On the other hand, a recently published study observed a strong correlation between maximal strength in front squat and deadlift and basketball-specific jump (*r* = 0.85–0.91) and sprint (*r* = –0.71–0.85) performance (8). Also, it has been found that performance on change of direction drills (i.e., T-test and 505) was strongly correlated with maximal dynamic, isometric, concentric as well as eccentric strength (*r* = –0.79–0.89) in female basketball players (11). Although focused on examining NCAA Division-I-AA football players, a statistically significant relationship was found between back squat 1RM adjusted by athlete's body mass and 40-yard (*r* = –0.61) and 10-yard (*r* = –0.54) sprint times (12), further solidifying the importance of strength as one of the key physical performance attributes. Additionally, the data collected over a span of seven years on elite NCAA Division-I basketball players revealed that lower-body strength was related to post-collegiate playing opportunities, with greater values being associated with higher levels of competitive play (2). Therefore, based on the amount of existing scientific literature, the importance of strength as one of the key physical performance attributes for optimizing on-court basketball performance should be undisputed.

Besides the physical performance component (e.g., strength, power, speed), basketball players need to possess and be proficient in executing sport-specific skills such as shooting, dribbling, passing, and rebounding. Among the aforementioned skills, previous research has found that shooting performance has the largest contribution to securing the winning game outcome on various levels of basketball competition (5, 13–16). For example, one of the recently published studies revealed that overall shooting efficiency (i.e., free-throw, two-point, and three-point) accounted for 23–26% of the total percentage of the explained variance when differentiating winning from losing game outcomes in the National Basketball Association (NBA) (5). Similar findings were observed by the same authors when examining games played on the NCAA Division-II competitive level (13). However, despite its importance, only a few research reports focused on examining the relationship between some of the fundamental physical performance attributes and basketball shooting efficiency (17–19). When studying a group of professional male basketball players, Pojksic et al. (18) observed that upper-body explosive power (i.e., medicine ball toss) was a good predictor of long-distance shooting performance during gameplay. In a follow-up investigation, the same group of authors found that jumping, throwing, and anaerobic endurance were good determinants of long-distance dynamic shooting performance (i.e., 60-sec timed star-pattern three-point shooting drill). In addition, superior upper-body and lower-body explosive power capacities were positively associated with three-point shooting efficiency (19). Contrary to the previously mentioned findings, Cabarkapa et al. (17) observed no significant relationship between maximal upper-body and lower-body strength in resistance-trained male and female basketball players. Neither bench press nor back squat 1RM was a good predictor of free-throw, two-point, and three-point shooting performance (17).

In a practical setting, individual and/or team training sessions are commonly arranged prior to on-court basketball practice to accommodate dense team schedules. With resistance exercise being widely implemented as a part of regular training regimens and shooting efficiency being one of the key basketball-specific skills that players need to possess, it is of critical importance for coaches, sports scientists, and strength and conditioning practitioners to recognize how they actually affect each other. Thus, to bridge a gap in the scientific literature, the purpose of the present study was to examine the acute impact of some of the most commonly implemented in-season upper-body and lower-body resistance training regimens on basketball shooting mechanics and accuracy.

## 2. Materials and methods

### 2.1. Participants

Ten resistance-trained recreationally active men with previous basketball playing experience (x̄ ± SD; height = 182.6 ± 9.7 cm; body mass = 79.2 ± 13.9 kg; age = 25.6 ± 5.5 years; organized playing experience = 9.5 ± 4.1 years) volunteered to participate in the present investigation. The inclusion criteria involved individuals capable of making ≥50% of the free-throw and two-point shots, and ≥30% of three-point shots during the familiarization session (Visit 1). All participants had ≥2 years of resistance training experience (4.8 ± 2.2 years) and actively participated in resistance training activities ≥2 times per week. Participants with current and/or previous musculoskeletal injuries that could potentially impair lifting and/or shooting performance were excluded from participation. All testing procedures performed in the present study were previously approved by the Institutional Review Board and all participants signed an informed consent document.

### 2.2. Procedures

**Figure 1:**
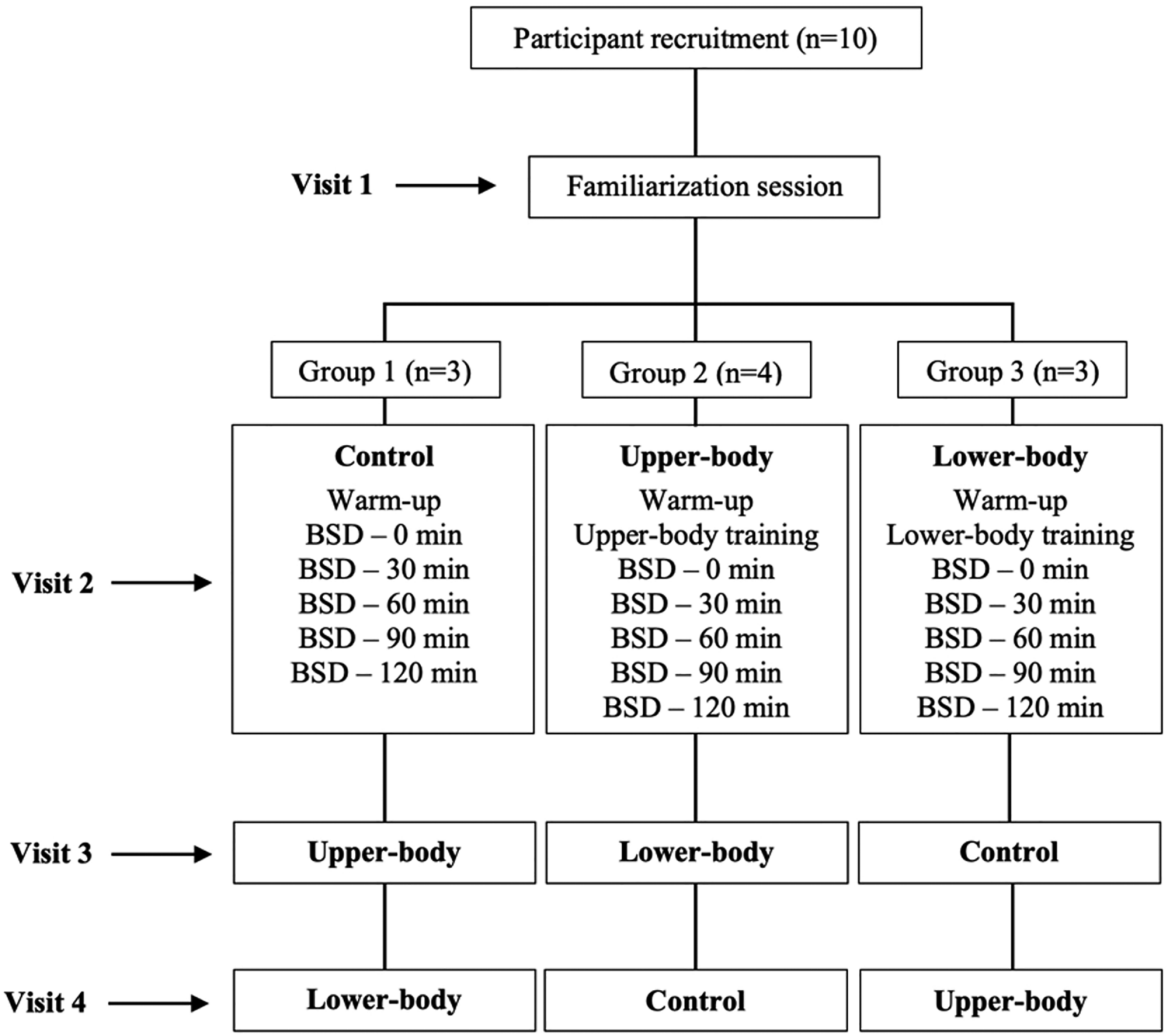
Graphical representation of testing procedures. BSD, basketball shooting drills.

All participants visited the laboratory on 4 different occasions. The initial visit (Visit 1) was dedicated to familiarizing participants with the overall testing procedures during which the inclusion criterium based on shooting proficiency was determined. If qualified, participants proceeded with the upper-body and lower-body 1RM testing protocols. Then, by using a random-number generator, each participant was assigned to one of the three testing groups (Groups 1–3). Each group performed control, upper-body, and lower-body treatments in randomized order (Visits 2–4). The control group performed the first set of basketball shooting drills immediately following the completion of a standardized dynamic warm-up protocol. The identical shooting protocol was repeated 4 more times, in 30 min increments. On the other hand, participants assigned to upper-body and lower-body treatment groups performed identical basketball shooting procedures upon completion of a specifically designed resistance training program. Each laboratory visit was separated 3–7 days apart to minimize the possible influence of fatigue. During each 30 min recovery period, participants stayed in the laboratory and actively rested. Also, participants were informed to maintain their regular physical activity levels and nutritional patterns as well as abstain from strenuous exercise (e.g., resistance training) throughout the course of the study. A detailed graphical representation of the experimental design is presented in Figure 1.

### 2.3. Basketball shooting protocol

**Figure 2:**
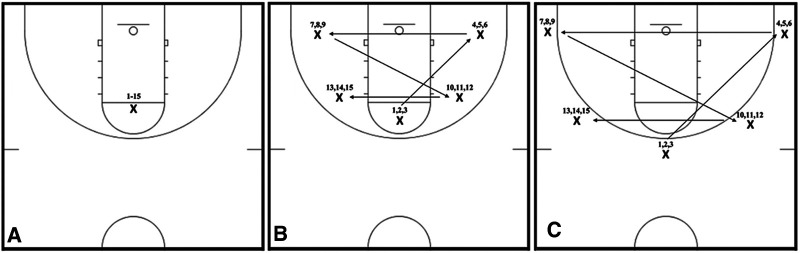
Graphical representation of free-throw (**A**), two-point (**B**), and three-point (**C**) shooting drills performed in the present investigation.

The basketball shooting drills used in the present study were based on Pojskic et al. (19) methodology. Free-throw, two-point, and three-point shots were attempted at 4.57, 5.18, and 6.75 m. Each block of basketball shooting drills, administered in 30 min increments, contained a total of 45 shots, from which 15 shots were attempted from each shooting distance. The number of shots attempted by each participant during a single laboratory visit was 225, and the overall number spread across 3 randomly ordered treatment modalities was 675. Free-throw shots were taken from the same spot (i.e., standardized free-throw line) presented in Figure 2A. Two-point shots were attempted from 5 different locations on the court, starting at the top of the key and following a star-pattern presented in Figure 2B (i.e., catch-and-shoot shots). Besides an increase in shooting distance, the three-point shooting drill presented in Figure 2C followed an identical movement pattern. Each shooting location was marked with white tape and a rubber cone to assure that participants attempted all shots from a consistent distance. Each block of 45 shots started exactly 30 min after the completion of the previous shooting session and/or resistance training protocol until the 120 min mark was reached (Visits 2–4).

To appropriately examine basketball shooting mechanics and accuracy without the presence of fatigue, the shooting drills were purposely designed to be stationary in nature. After completing 3 shots at the designated two-point or three-point shooting distances, the participant walked over to the next shooting location. Each set of 15 shots within a single shooting session was separated by a 2 min rest interval. A rebounder was present throughout the whole testing procedure and no other players were allowed on the court. All shooting procedures were performed on an indoor hardwood basketball court. The basket height (3.05 m) and the ball size used in this study corresponded to the official game regulations standards (i.e., Size 7, 74.9 cm, 624 g).

### 2.4. Biomechanical variables

The biomechanical parameters examined in the present study are based on previously published research reports, including a continuous line of research pertaining to basketball shooting mechanics conducted in our laboratory (20–28). The following variables were analyzed during the preparatory phase of the shooting motion (i.e., initial upward/concentric movement while the shooter was still on the ground): knee angle (i.e., internal angle between thigh and shank), ankle angle (i.e., angle between shank and an imaginary line parallel to the ground), hip angle (i.e., internal angle between the torso and thigh), shoulder angle (i.e., internal angle between the upper arm and torso), elbow angle (i.e., internal angle between upper arm and forearm), and elbow height (i.e., perpendicular distance between the olecranon process and the ground divided by the participant's body height).

**Figure 3:**
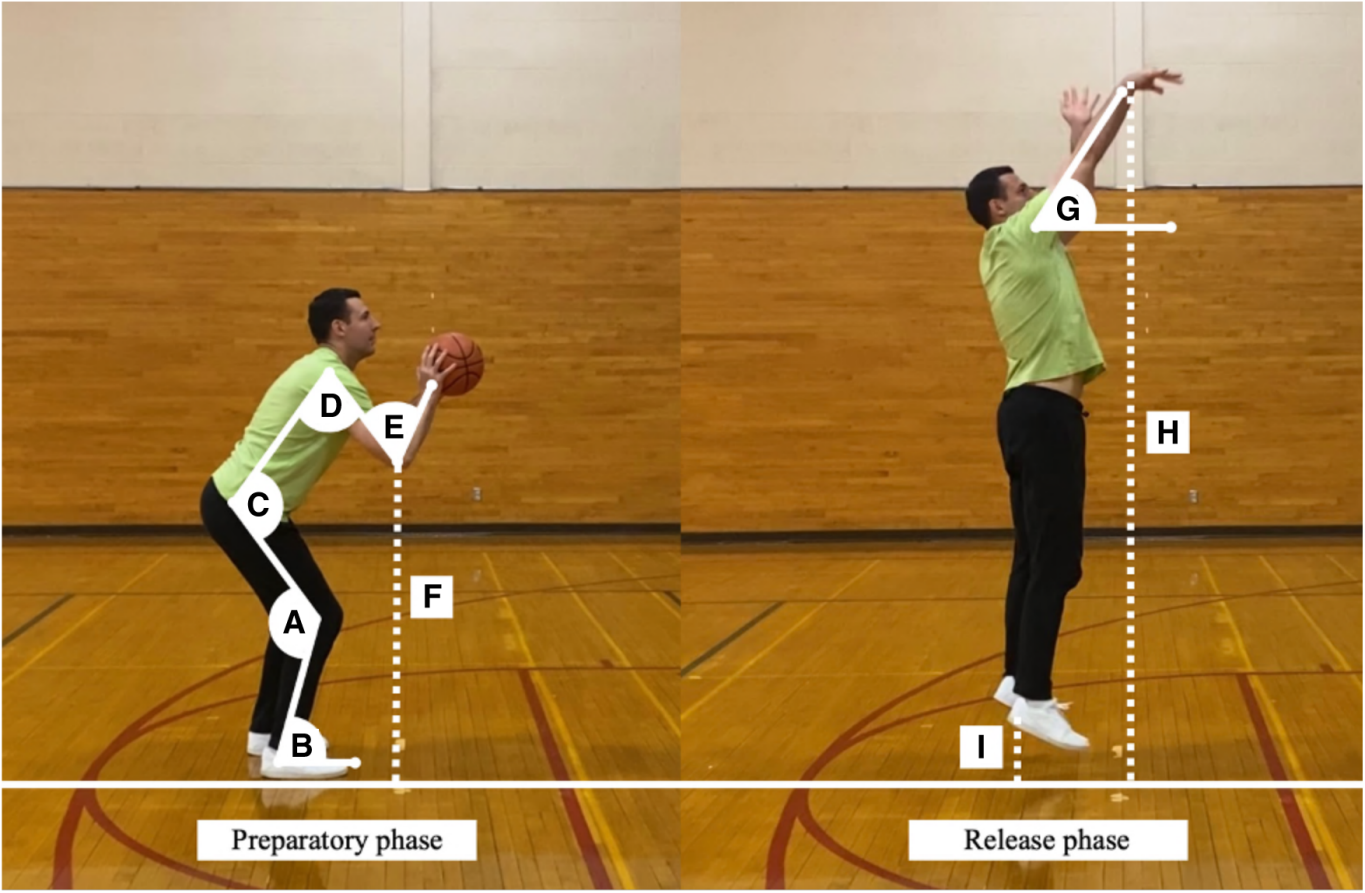
Graphical representation of the biomechanical parameters examined during the preparatory and release phases of shooting motions. Knee angle (**A**); ankle angle (**B**); hip angle (**C**); shoulder angle (**D**); elbow angle (**E**); elbow height (**F**); release angle (**G**); release height (**H**); heel height (**I**).

In a similar manner, kinematic variables examined at the release phase of the shooting motion (i.e., time point when the ball left the shooter's hand) were: release angle (i.e., angle between the fully extended arm and an imaginary line parallel to the ground), release height (i.e., perpendicular distance from the center of the ball to the ground divided by the participant's body height), heel height (i.e., perpendicular distance from the calcaneus to the ground). In addition, the kinematic variable related to the ball trajectory examined this study was the entry angle (i.e., angle at which the ball entered the rim). The graphical representation of biomechanical parameters examined in this investigation is presented in Figure 3.

Kinematic variables during both preparatory and release phases of the shooting motion were derived from a high-definition high-speed camera (Sony Cyber-Shot DCS-RX10 III, Tokyo, Japan) recording at 120 fps positioned 10 m away perpendicular to the participant's shooting plane of motion (i.e., shooting-hand side). Only the first 3 shots taken at the top of the key (i.e., center-court shooting position) for each of the shooting drills examined in the present study (i.e., free-throw, two-point, and three-point) were video recorded and used for kinematic analysis purposes (i.e., 9 shots per shooting session, 45 total shots per visit). All kinematic variables were derived post-data collection by video analysis software (Kinovea, Version 0.9.3). In addition, an innovative basketball tracking system (Noah Basketball, Athens, AL, USA) mounted directly over the rim near the ceiling was used to wirelessly obtain the entry angle (i.e., the angle at which the ball entered the rim) as well as to track all attempted and made shots from each shooting position during the course of the study.

### 2.5. Resistance exercise stimulus

Prior to the start of both upper-body and lower-body resistance training protocols, all participants performed a standardized dynamic warm-up consisting of a five-minute treadmill run (Life Fitness 95 T, IL, USA) at a self-selected moderate intensity and a set of dynamic stretching exercises (e.g., butt-kicks, A-skips, side-to-side lunges, quad pulls). Along with close consultation with collegiate and professional basketball strength and conditioning practitioners, both resistance training regimens were developed based on the National Strength and Conditioning Association guidelines (29). While capable of eliciting further improvements in strength and power, the primary purpose of these training programs was directed toward maintaining previously attained performance gains in strength and power obtained during pre-season competitive period. The load, repetitions, and the number of sets were carefully selected to mimic the in-season training program commonly implemented with basketball players (29). To assure adequate recovery, the rest interval between each exercise set was 90–120 sec. The upper-body and lower-body resistance training plan is presented in Table 1.

**Table 1:** Upper-body and lower-body resistance training protocols.

Upper-body resistance training session
Push press — 1 × 3 (75% 1RM), 1 × 3 (80% 1RM), 1 × 3 (85% 1RM)
Barbell bench press — 1 × 8 (75% 1RM), 1 × 6 (80% 1RM), 1 × 4 (85% 1RM)
Barbell bent-over row — 1 × 8 (75% 1RM), 1 × 6 (80% 1RM), 1 × 4 (85% 1RM)
Lateral shoulder raise — 3 × 10 RM
Dumbbell biceps curls — 3 × 10 RM (each arm)
Triceps cable extensions — 3 × 10 RM
Lower-body resistance training session
Mid-thigh hang power clean — 1 × 3 (75% 1RM), 1 × 3 (80% 1RM), 1 × 3 (85% 1RM)
Barbell back squat — 1 × 8 (75% 1RM), 1 × 6 (80% 1RM), 1 × 4 (85% 1RM)
Trap bar deadlift — 1 × 8 (75% 1RM), 1 × 6 (80% 1RM), 1 × 4 (85% 1RM)
Dumbbell lunges — 3 × 10 RM (each leg)
Single leg Romanian dead lifts — 3 × 10 RM

1RM, one-repetition maximum; RM, repetition maximum.

### 2.6. One-repetition maximum testing

When testing 1RM for a barbell bench press exercise, the participants completed 5–10 repetitions of light to moderate weight (i.e., 40–50% of their estimated 1RM), then performed two heavier sets of 3–5 repetitions (i.e., 60–80% of their estimated 1RM). In 2–3-min increments, the weight was increased by 5–10% for each lift that the participant successfully completed. If unable to complete the lift while maintaining the proper lifting technique, the weight was reduced until the maximum amount of weight that the participant was capable of lifting was reached (11). An identical testing procedure was implemented for determining 1RM for a barbell back squat exercise. On the other hand, the 1RM for push press, bent-over row, mid-thigh hang power clean, and trap bar deadlift were estimated based on velocity-based training guidelines from submaximal barbell velocity measurements assessed via a three-dimensional camera-based system (Elite Form, Lincoln, NE, USA) during the familiarization visit (Visit 1) (30, 31). The repetition maximum for the rest of the exercises used in this study were estimated based on the participant's previous lifting experience.

### 2.7. Statistical analysis

All statistical analyses were performed using R software (Version 4.2.1; R Foundation for Statistical Computing, Vienna, Austria). To determine the effect of condition and time on each dependent variable, for each type of shooting motion (i.e., free-throw, two-point, three-point), separate lme4 restricted maximum likelihood linear mixed-effects models were fitted (32). Condition and time were specified as model-fixed effects and participant as a random effect. Assumptions of residual normality and homoscedasticity were visually verified using Q-Q plots and model-predicted scores vs. residuals plots, respectively. Fixed effects were analyzed for significant main effects through F tests using Satterthwaite's method of estimating denominator degrees of freedom (32). Fixed effect *p*-values were obtained using lmerTest (33). When necessary, pairwise comparisons were carried out using estimated marginal means with Hommel's multiple comparison *p*-value correction methods (34). Alpha was set at *a priori* at *p* < 0.05. Fixed effect sizes were quantified as semi-partial R2 values obtained using the Nakagawa et al. (35) approach via r2beta in r2glmm (36).

## 3. Results

For the free-throw shooting motion, a statistically significant interaction was observed between condition and time (*p* = 0.024) in shooting percentage. Follow-up contrasts showed pairwise differences within the first testing timepoint and no differences within any other testing timepoints. The lower-body condition exhibited a significantly higher shooting percentage than the upper-body condition (*p* < 0.001; CI: 0.06–0.25), while no difference was found when compared to the control condition (*p* = 0.057; CI: −0.19–0.01), nor was there any significant differences observed between control and upper-body conditions (*p* = 0.117; CI: −0.04–0.16).

For the two-point shooting motion, no statistically significant interaction effect was observed (*p* = 0.870). Also, no significant main effect for time was detected (*p* = 0.850). However, there was a statistically significant main effect of the condition (*p* = 0.023). Follow-up contrasts revealed a difference between control and upper-body conditions, where the shooting percentage experienced an 11.8% mean decrease immediately following the upper-body resistance training session (*p* = 0.019; CI: 0.01–0.14). No other pairs of conditions were significantly different.

For the three-point shooting motion, no statistically significant interaction effect between condition and time was observed (*p* = 0.960). However, there was a significant main effect of condition (*p* < 0.001) where the upper-body training session led to a significant decrease in shooting percentage when compared to both control (*p* < 0.001; CI: 0.04–0.14) and lower-body conditions (*p* = 0.011; CI: 0.01–0.11), 9.9% and 9.3%, respectively. Also, despite being very small in magnitude, there was a significant main effect of time (*p* = 0.030) observed, where the shooting percentage at the last testing timepoint increased when compared to the first timepoint (*p* = 0.014; CI: 0.01–0.17). No other combinations of testing timepoints were significantly different.

Interestingly, while statistically significant main effects were detected in elbow height, heel height, and shoulder angle for the free-throw shooting motion, and ankle angle, elbow height, and entry angle for three-point shooting motion, the magnitudes of these semi-partial R^2^ values were extremely small. Moreover, for the majority of kinematic variables of free-throw, two-point, and three-point shooting motions, the combination of all fixed effects could account for <1% of the total variance in each kinematic variable. Thus, they will not be highlighted. The means and standard deviations (x̄ ± SD) and effect sizes for each variable are reported in Tables 2–4.

**Table 2:** Descriptive statistics for biomechanical parameters examined during the preparatory phase of the free-throw, two-point, and three-point shooting motions.

	Time [min]	Free-throw			Two-point			Three-point		
		Control	Lower-body	Upper-body	Control	Lower-body	Upper-body	Control	Lower-body	Upper-body
Ankle angle [deg]	0	50.8 ± 8.1	51.0 ± 6.8	50.8 ± 8.7	49.4 ± 6.2	50.4 ± 6.2	49.5 ± 7.5	46.5 ± 6.2	46.9 ± 7	47.3 ± 7.3
	30	50.7 ± 7.1	51.4 ± 6.8	51.3 ± 7.3	48.8 ± 6.0	50.6 ± 6.6	48.3 ± 7.5	45.6 ± 7.6	47.5 ± 7.2	46.5 ± 7.4
	60	51.1 ± 6.7	51.3 ± 7.1	51.6 ± 7.9	48.9 ± 6.5	49.5 ± 6.1	49.9 ± 7.0	46.1 ± 7.4	46.2 ± 7.5	46.8 ± 7.3
	90	51.1 ± 7.2	49.8 ± 6.9	50.3 ± 9.2	49.7 ± 6.3	49.1 ± 5.8	49.0 ± 6.2	45.5 ± 7.8	46.4 ± 7.7	46.3 ± 7.9
	120	50.6 ± 7.6	51.2 ± 7.5	50.8 ± 7.1	49.4 ± 6.5	49.5 ± 5.7	49.8 ± 5.2	45.0 ± 7.1	45.0 ± 7.3	45.7 ± 6.6
Knee angle [deg]	0	106.6 ± 14.8	106.7 ± 11.8	106.7 ± 17.0	108.2 ± 9.9	105 ± 12.3	106.6 ± 11.7	103.6 ± 8.9	103.6 ± 8.2	105.2 ± 10.5
	30	107.4 ± 13.0	107.2 ± 13.3	107.1 ± 13.5	107.2 ± 8.3	108.7 ± 8.3	105.7 ± 11.8	103.1 ± 10.5	105.4 ± 9.7	103.5 ± 10.3
	60	106.7 ± 12.2	107.1 ± 13.2	107.2 ± 14.5	106.9 ± 9.1	108.2 ± 7.8	107.5 ± 11.5	103.4 ± 10.7	103.4 ± 9.4	104.4 ± 10.5
	90	108.4 ± 12.4	104.7 ± 14.3	107.3 ± 14.2	108.5 ± 8.8	107.4 ± 8.4	106.8 ± 9.8	103.5 ± 10.9	103.2 ± 8.8	103.7 ± 10.6
	120	106.7 ± 13.6	107 ± 13.6	106.9 ± 14.6	107.4 ± 9.1	107.8 ± 8.4	107.8 ± 8.2	103.0 ± 10.0	102.1 ± 9.7	103.0 ± 8.3
Hip angle [deg]	0	133.9 ± 16.7	133.5 ± 14.8	134.7 ± 17.9	139.7 ± 7.0	137.9 ± 6.7	138.2 ± 9.1	133.5 ± 8.5	132.3 ± 5.8	133.2 ± 9.4
	30	134.5 ± 17.3	134.8 ± 17.4	135.2 ± 17.0	138.4 ± 8.4	138.8 ± 8.7	137.7 ± 8.7	133.8 ± 7.7	134.3 ± 7.0	133.2 ± 8.4
	60	133.6 ± 15.7	134.4 ± 16.4	136.0 ± 18.3	138.5 ± 8.1	140.4 ± 6.9	139.6 ± 8.0	133.5 ± 7.4	133.5 ± 8.5	134.6 ± 8.7
	90	136.0 ± 14.4	133.4 ± 17.0	135.6 ± 15.6	139.1 ± 8.0	139.3 ± 6.5	138.5 ± 8.0	133.8 ± 7.9	133.2 ± 6.6	133.7 ± 8.9
	120	133.6 ± 15.9	135.1 ± 15.3	134.5 ± 16.3	138.5 ± 7.4	138.5 ± 8.0	139.1 ± 6.4	135.2 ± 7.3	133.9 ± 7.3	132.9 ± 7.1
Shoulder angle [deg]	0	79.1 ± 19.9	82.2 ± 18.1	79.0 ± 17.6	84.4 ± 13.8	83.8 ± 15.9	81.6 ± 14.4	76.2 ± 17.9	75.9 ± 17.9	74.3 ± 18.9
	30	80.0 ± 20.5	78.2 ± 18.2	78.6 ± 17.7	83.5 ± 13.1	82.2 ± 15.3	87.0 ± 16.3	76.2 ± 17.3	76.8 ± 15.9	76.0 ± 17.2
	60	76.5 ± 22.7	76.6 ± 16.5	78.9 ± 18.2	84.2 ± 14.1	81.7 ± 13.1	83.3 ± 14.1	76.9 ± 15.3	73.1 ± 15.0	75.3 ± 16.4
	90	77.6 ± 22.3	75.7 ± 18.9	76.6 ± 18.4	82.9 ± 15.3	80.3 ± 12.6	80.3 ± 11.9	76.2 ± 16.2	74.3 ± 15.7	72.8 ± 15.5
	120	76.8 ± 24.3	75.3 ± 17.2	75.8 ± 17.5	82.6 ± 13.0	81.2 ± 12.6	80.8 ± 12.2	74.9 ± 17.3	72.9 ± 15.2	74.3 ± 16.5
Elbow angle [deg]	0	57.9 ± 9.3	57.2 ± 8.2	57.9 ± 8.7	57.9 ± 11.8	57.5 ± 10.1	57.5 ± 10.8	56.0 ± 11.9	56.8 ± 11.1	56.6 ± 12.9
	30	57.4 ± 11.5	57.8 ± 7.7	57.9 ± 10.3	58.4 ± 11.0	58 ± 10.4	57.7 ± 12.2	55.8 ± 12.2	56.7 ± 11.0	56.2 ± 11.8
	60	59.0 ± 12.3	57.7 ± 10.4	57.7 ± 9.5	57.9 ± 12.0	57.4 ± 11.8	57.4 ± 11.4	56.5 ± 12.6	55.9 ± 10.6	55.4 ± 11.2
	90	58.4 ± 11.5	59.2 ± 8.2	59.6 ± 10.7	58.6 ± 10.4	57.9 ± 10.2	57.8 ± 9.1	55.0 ± 9.1	56.4 ± 11.3	56.3 ± 10.6
	120	58.8 ± 10.2	58.8 ± 8.4	59.7 ± 10.1	58.2 ± 11.4	57.4 ± 9.4	57.7 ± 10.2	57.3 ± 12.1	56.1 ± 9.6	57.5 ± 10.5
Elbow height [ratio]	0	0.65 ± 0.10	0.66 ± 0.09	0.66 ± 0.11	0.68 ± 0.06	0.67 ± 0.06	0.66 ± 0.06	0.63 ± 0.07	0.62 ± 0.07	0.62 ± 0.07
	30	0.67 ± 0.11	0.65 ± 0.09	0.65 ± 0.13	0.68 ± 0.05	0.67 ± 0.05	0.67 ± 0.06	0.62 ± 0.06	0.63 ± 0.06	0.63 ± 0.07
	60	0.66 ± 0.09	0.64 ± 0.10	0.65 ± 0.10	0.68 ± 0.04	0.67 ± 0.04	0.67 ± 0.10	0.63 ± 0.07	0.61 ± 0.05	0.62 ± 0.07
	90	0.66 ± 0.10	0.64 ± 0.09	0.65 ± 0.09	0.68 ± 0.05	0.67 ± 0.05	0.66 ± 0.05	0.63 ± 0.07	0.61 ± 0.05	0.61 ± 0.06
	120	0.66 ± 0.10	0.65 ± 0.08	0.65 ± 0.10	0.67 ± 0.05	0.67 ± 0.05	0.67 ± 0.05	0.62 ± 0.06	0.60 ± 0.06	0.62 ± 0.07

**Table 3:** Descriptive statistics for biomechanical parameters examined during the release phase of the free-throw, two-point, and three-point shooting motions.

	Time [min]	Free-throw			Two-point			Three-point		
		Control	Lower-body	Upper-body	Control	Lower-body	Upper-body	Control	Lower-body	Upper-body
Release angle [deg]	0	56.5 ± 6.4	57.6 ± 6.3	56.2 ± 6.5	55.2 ± 7.1	56.1 ± 7.3	54.8 ± 6.9	50.7 ± 6.3	50.7 ± 7.0	50.1 ± 5.9
	30	57.1 ± 5.6	57.6 ± 6.1	57.3 ± 6.7	55.0 ± 6.7	55.3 ± 6.3	54.8 ± 6.2	50.4 ± 6.1	51.0 ± 6.4	51.0 ± 6.5
	60	56.6 ± 5.7	57.4 ± 5.7	57.2 ± 6.1	55.5 ± 5.6	55.4 ± 7.5	55.7 ± 5.5	50.8 ± 6.2	51.5 ± 7.2	51.4 ± 6.9
	90	56.9 ± 5.2	57.2 ± 6.5	57.3 ± 5.8	55.3 ± 5.5	55.5 ± 6.9	55.7 ± 6.7	50.3 ± 5.9	51.1 ± 7.6	49.9 ± 7.1
	120	57.1 ± 5.3	56.7 ± 5.8	56.9 ± 5.9	55.4 ± 5.1	55.4 ± 6.8	54.8 ± 6.4	50.6 ± 6.3	51.4 ± 6.5	50.6 ± 7.3
Release height [ratio]	0	1.27 ± 0.04	1.27 ± 0.05	1.28 ± 0.04	1.30 ± 0.05	1.29 ± 0.03	1.30 ± 0.05	1.29 ± 0.04	1.27 ± 0.08	1.30 ± 0.05
	30	1.27 ± 0.03	1.27 ± 0.03	1.24 ± 0.11	1.30 ± 0.05	1.29 ± 0.04	1.26 ± 0.13	1.29 ± 0.05	1.30 ± 0.05	1.30 ± 0.04
	60	1.28 ± 0.04	1.26 ± 0.04	1.27 ± 0.04	1.30 ± 0.04	1.30 ± 0.05	1.30 ± 0.05	1.30 ± 0.08	1.29 ± 0.05	1.27 ± 0.10
	90	1.27 ± 0.04	1.27 ± 0.05	1.28 ± 0.04	1.29 ± 0.05	1.29 ± 0.05	1.30 ± 0.04	1.29 ± 0.05	1.28 ± 0.05	1.28 ± 0.06
	120	1.28 ± 0.04	1.28 ± 0.04	1.28 ± 0.04	1.30 ± 0.05	1.29 ± 0.06	1.29 ± 0.05	1.29 ± 0.05	1.27 ± 0.07	1.29 ± 0.05
Heel height [cm]	0	12.7 ± 3.4	12.9 ± 3.4	12.9 ± 3.7	20.9 ± 7.0	19.9 ± 6.1	20.7 ± 6.1	25.6 ± 4.5	24.8 ± 4.7	25.3 ± 4.6
	30	13.1 ± 3.7	12.8 ± 2.7	11.9 ± 3.8	20.7 ± 6.3	20.1 ± 5.3	20.0 ± 5.7	25.4 ± 4.4	25.0 ± 4.4	24.9 ± 4.7
	60	13.6 ± 3.5	13.3 ± 2.6	12.0 ± 3.4	20.6 ± 7.0	20.4 ± 5.2	19.5 ± 5.7	25.3 ± 4.5	25.0 ± 5.0	24.2 ± 4.8
	90	13.0 ± 3.1	13.1 ± 3.0	12.3 ± 3.4	20.3 ± 6.2	20.5 ± 5.6	20.4 ± 5.4	25.1 ± 4.0	24.9 ± 4.6	24.5 ± 5.0
	120	13.2 ± 3.2	13.1 ± 2.8	12.8 ± 3.3	20.1 ± 6.3	20.1 ± 6.4	19.7 ± 5.6	25.2 ± 4.2	25.2 ± 4.1	25.1 ± 5.6
Entry angle [deg]	0	41.4 ± 3.6	43.8 ± 4.2	42.3 ± 3.7	44.2 ± 3.5	45.0 ± 2.8	44.5 ± 2.2	45.4 ± 2.8	46.8 ± 2.2	46.2 ± 2.9
	30	43.5 ± 4.1	43.1 ± 3.7	43.7 ± 4.6	44.8 ± 3.9	44.4 ± 3.3	43.9 ± 3.3	45.9 ± 2.9	46.4 ± 3.6	45.4 ± 1.7
	60	43.1 ± 4.6	43.5 ± 3.9	43.7 ± 3.9	44.7 ± 3.3	44.4 ± 3.0	44.0 ± 2.8	45.6 ± 3.2	46.3 ± 3.5	46.3 ± 1.6
	90	42.9 ± 4.2	43.2 ± 4.4	43.8 ± 4.2	44.7 ± 2.9	44.8 ± 3.9	44.3 ± 2.9	45.6 ± 3.5	46.2 ± 3.1	46.0 ± 2.3
	120	43.4 ± 4.5	43.7 ± 4.1	42.9 ± 3.9	44.6 ± 3.2	44.2 ± 3.2	44.6 ± 3.3	45.8 ± 3.6	46.6 ± 3.0	45.6 ± 2.5
Shooting percentage [%]	0	68.5 ± 19.0	77.5 ± 8.8	62.1 ± 15.4	68.5 ± 18.4	61.4 ± 17.9	56.7 ± 20.6	48.6 ± 21.0	48.0 ± 16.8	38.7 ± 18.5
	30	75.4 ± 17.6	75.4 ± 14.4	74.6 ± 16.0	64.6 ± 12.6	64.7 ± 14.4	57.4 ± 18.7	52.0 ± 14.3	49.9 ± 17.2	44.7 ± 20.1
	60	78.7 ± 8.7	75.1 ± 16.1	77.3 ± 12.6	66.6 ± 23.4	67.2 ± 17.9	61.9 ± 24.4	54.0 ± 14.3	48.6 ± 15.0	45.5 ± 15.1
	90	75.3 ± 14.4	73.2 ± 18.9	79.3 ± 14.4	65.2 ± 14.0	65.8 ± 14.0	62.7 ± 20.8	54.7 ± 18.3	49.2 ± 22.7	41.5 ± 17.6
	120	74.1 ± 8.5	76.6 ± 14.5	78.8 ± 13.6	70.7 ± 12.6	60.4 ± 15.3	60.8 ± 16.5	56.0 ± 18.7	56.0 ± 18.4	50.8 ± 12.2

**Table 4:** Fixed effect semi-partial *R*^2^ effect sizes with confidence interval lower-limits (LL) and upper-limits (UL) for all dependent variables during preparatory and release phases of the free-throw, two-point, and three-point shooting motions.

	Fixed effects	Free-throw			Two-point			Three-point		
		*R* ^2^	*R*^2^ LL	*R*^2^ UL	*R* ^2^	*R*^2^ LL	*R*^2^ UL	*R* ^2^	*R*^2^ LL	*R*^2^ UL
Ankle angle	All	0.003	0.037	0.160	0.008	0.039	0.166	0.010	0.039	0.168
	Condition*Time	0.002	0.015	0.112	0.005	0.016	0.118	0.002	0.015	0.112
	Condition	0.000	0.000	0.048	0.001	0.000	0.052	**0** **.** **000**	**0** **.** **000**	**0** **.** **050**
	Time	0.000	0.003	0.072	0.001	0.003	0.074	**0** **.** **002**	**0** **.** **003**	**0** **.** **075**
Knee angle	All	0.003	0.037	0.159	0.010	0.040	0.169	0.007	0.038	0.165
	Condition*Time	0.002	0.015	0.113	0.008	0.016	0.122	0.003	0.015	0.115
	Condition	0.000	0.000	0.048	0.004	0.000	0.060	0.001	0.000	0.052
	Time	0.001	0.003	0.074	0.001	0.003	0.075	0.000	0.003	0.072
Hip angle	All	0.003	0.037	0.159	0.008	0.039	0.166	0.007	0.038	0.165
	Condition*Time	0.002	0.015	0.112	0.005	0.016	0.118	0.005	0.015	0.117
	Condition	0.000	0.000	0.049	0.002	0.000	0.055	0.001	0.000	0.051
	Time	0.001	0.003	0.074	0.001	0.003	0.075	0.002	0.003	0.077
Shoulder angle	All	0.009	0.039	0.167	0.016	0.042	0.176	0.007	0.038	0.164
	Condition*Time	0.003	0.015	0.114	0.007	0.016	0.121	0.002	0.015	0.113
	Condition	0.001	0.000	0.052	0.002	0.000	0.053	0.001	0.000	0.050
	Time	**0** **.** **002**	**0** **.** **003**	**0** **.** **076**	0.001	0.003	0.073	0.000	0.003	0.073
Elbow height	All	0.006	0.038	0.163	0.011	0.040	0.170	0.014	0.041	0.174
	Condition*Time	0.003	0.015	0.114	0.003	0.015	0.114	0.004	0.015	0.116
	Condition	**0** **.** **000**	**0** **.** **000**	**0** **.** **049**	0.004	0.000	0.061	**0** **.** **001**	**0** **.** **000**	**0** **.** **053**
	Time	0.001	0.003	0.073	0.001	0.003	0.074	0.001	0.003	0.073
Elbow angle	All	0.006	0.038	0.164	0.001	0.036	0.157	0.003	0.037	0.160
	Condition*Time	0.002	0.015	0.112	0.000	0.014	0.109	0.002	0.015	0.112
	Condition	0.000	0.000	0.049	0.000	0.000	0.049	0.000	0.000	0.049
	Time	0.001	0.003	0.075	0.000	0.003	0.072	0.002	0.003	0.075
Release angle	All	0.004	0.037	0.161	0.003	0.037	0.160	0.005	0.037	0.162
	Condition*Time	0.002	0.015	0.113	0.002	0.015	0.112	0.001	0.015	0.111
	Condition	0.002	0.000	0.055	0.001	0.000	0.053	0.000	0.000	0.049
	Time	0.001	0.003	0.073	0.000	0.003	0.072	0.000	0.003	0.072
Release height	All	0.042	0.054	0.208	0.027	0.046	0.190	0.026	0.046	0.189
	Condition*Time	0.018	0.020	0.138	0.014	0.018	0.131	0.014	0.018	0.133
	Condition	0.001	0.000	0.053	0.000	0.000	0.049	0.007	0.001	0.067
	Time	0.002	0.003	0.076	0.001	0.003	0.074	0.001	0.003	0.074
Heel height	All	0.018	0.043	0.179	0.004	0.037	0.161	0.006	0.038	0.163
	Condition*Time	0.006	0.016	0.120	0.002	0.015	0.113	0.002	0.015	0.113
	Condition	**0** **.** **000**	**0** **.** **000**	**0** **.** **049**	0.001	0.000	0.052	0.001	0.000	0.052
	Time	0.003	0.004	0.078	0.001	0.003	0.073	0.001	0.003	0.073
Entry angle	All	0.023	0.045	0.185	0.008	0.039	0.167	0.022	0.044	0.184
	Condition*Time	0.012	0.018	0.129	0.005	0.016	0.118	0.007	0.016	0.121
	Condition	0.012	0.001	0.079	0.002	0.000	0.055	**0** **.** **007**	**0** **.** **001**	**0** **.** **068**
	Time	0.012	0.005	0.096	0.002	0.003	0.075	0.001	0.003	0.074
Shooting percentage	All	0.080	0.073	0.248	0.044	0.055	0.210	0.073	0.070	0.242
	Condition*Time	0.046	0.031	0.177	0.014	0.018	0.132	0.006	0.016	0.120
	Condition	0.036	0.004	0.123	**0** **.** **015**	**0** **.** **001**	**0** **.** **085**	**0** **.** **013**	**0** **.** **001**	**0** **.** **082**
	Time	**0** **.** **017**	**0** **.** **006**	**0** **.** **106**	0.005	0.004	0.084	**0** **.** **007**	**0** **.** **004**	**0** **.** **088**

Bolded values indicate statistically significant effects (*p* < 0.05).

## 4. Discussion

To the best of our knowledge, this is the first study to examine the acute impact of resistance exercise on shooting mechanics and accuracy in male basketball players. Our results indicate that 10 selected biomechanical parameters during preparatory and release phases of free-throw, two-point, and three-point shooting motions remain unchanged following both upper-body and lower-body resistance training protocols. The combination of all fixed effects could account for <1% of the total variance in each dependent variable pertaining to basketball shooting mechanics. On the other hand, despite biomechanical characteristics remaining unchanged throughout the testing protocol, the upper-body resistance training session provoked a 9.9–11.8% decrease in two-point and three-point shooting accuracy when compared to the control condition. However, the observed decline in shooting performance was only present immediately upon completion of the upper-body resistance training session (i.e., 0 min). The observed decrease in performance disappeared following a second set of basketball shooting drills (i.e., 30 min) and remained absent throughout the rest of the testing period (i.e., 120 min).

Previous research conducted on a cohort of NCAA Division-I women's basketball players revealed that free-throw and two-point speed spot shooting accuracy (i.e., attempt as many shots as possible within 60 sec period), alongside vertical jump height and anaerobic power, remained unchanged 6 h following a bout of a resistance training exercise (37). Regardless of the differences in the testing timeline and resistance training regimen design that involved a mix of upper-body and lower-body strength and power exercises (e.g., hang clean, push jerk, bench press, back squat), these findings are similar to the results obtained in the present investigation. No significant differences in free-throw, two-point and three-point shooting accuracy were observed 120 min post-completion of either upper-body and/or lower-body resistance training sessions, and it is likely that these observations were to remain unchanged if shooting protocols were to be completed at the 6-hour time mark. Nevertheless, it is important to note that the shooting protocols implemented in the present study were purposely designed to be non-fatiguing in nature. An increase in heart rate resulting from an increase in the physiological and metabolic demands of competitive gameplay has been shown to elicit a notable decrease in shooting accuracy (38). In a study focused on examining a group of resistance-trained semiprofessional male basketball players with >5 years of competitive experience, Freitas et al. (39) found that high resistance circuit training caused a 9.4% decrease in three-point shooting performance when compared to resting conditions (i.e., 48.3% vs. 38.9%). These findings are contradictory to our results regarding the three-point shooting accuracy post-lower-body training session, where no significant differences were observed when compared to the control condition, but almost identical in magnitude following the upper-body resistance training protocol (i.e., 0 min). While the exercise selection (e.g., half-squat vs. back squat) and rest interval (i.e., 40 sec vs. 90–120 sec) could be contributing factors for the observed discrepancies, these findings may lead us to assume that performing upper-body resistance exercise may have a greater impact on long-distance shooting performance than solely performing lower-body or mix of upper-body and lower-body resistance exercises (e.g., full body circuit training). Still, it should be noted that this suppression in shooting accuracy for both two-point and three-point shooting motions was non-existent 30 min post training session, suggesting that this decline in performance may not be of critical importance in a practical setting, especially since resistance training sessions are not scheduled prior to an official game.

Another interesting finding observed in the present study is that neither upper-body nor lower-body resistance training regimens produced significant changes in free-throw, two-point, and three-point shooting mechanics. Moreover, all biomechanical parameters during both the preparatory and release phases of the shooting motion remained consistent across five testing timepoints (i.e., 0–120 min). When compared to previously conducted research reports, our results are similar in magnitude to a group of proficient basketball shooters (21, 22, 24, 40, 41), which is expected considering that participants who volunteered to participate in the present study had a considerable amount of previous basketball playing experience (i.e., 9.5 ± 4.1 years). Yet, it is important to mention that despite a detailed analysis of the kinematics of shooting motion, some of the key biomechanical parameters such as movement velocity (e.g., hip and knee mean and peak angular velocities), have not been examined in this investigation (26, 27, 41). Lower release velocities were found to be related to greater shooting accuracy as they decrease body segmental movement variability and improve the consistency of the shooting motion (26, 27). Although kinematic parameters assessed in this investigation fall within the desired ranges observed within proficient basketball shooters during both the preparatory and release phases of the shooting motion (21, 22, 24, 40, 41), the movement velocity could have been acutely altered (i.e., 0 min) during the transition phase (i.e., start to end of the shooting motion). While further research is warranted on this topic, this could potentially explain suppression in two-point and three-point shooting accuracy observed immediately following the completion of upper-body and lower-body resistance training protocols. In addition, this assumption can be supported by recently published data from our laboratory obtained via an innovative three-dimensional markerless motion capture system indicating that lower knee peak and mean angular velocities were related to superior free-throw shooting accuracy (42).

While these findings allow coaches, sports scientists, and strength and conditioning practitioners to obtain a deeper insight into the acute influence of resistance training on basketball shooting mechanics and accuracy, this study is not without limitations. The testing procedures were conducted in a controlled laboratory-based setting that does not directly mimic in-game competitive requirements and the sample size could have been larger. Also, the biomechanical analysis included only initial three shoots attempts at the mid-court position (e.g., top of the key) for each shooting motion, which may limit the applicability of these findings to other locations on the court. Future research needs to examine if these findings remain applicable across various levels of basketball competition (e.g., collegiate, professional) and if they are gender-specific. In addition, considering the exponential growth, good reliability, and practical applicability of markerless motion capture systems, future research should consider implementing this type of technology for the assessment of various biomechanical characteristics of basketball sport-specific motions during live gameplay.

In conclusion, the findings of the present study indicate that some of the most commonly implemented basketball-specific upper-body and lower-body resistance exercise regimens during the in-season competitive period (i.e., maintenance phase) have minimal to no impact on free-throw, two-point and three-point kinematics during preparatory and release phases of the shooting motion. On the other hand, it should be noted that the upper-body resistance training session induced an acute suppression in two-point and three-point shooting accuracy (i.e., 9.9–11.8%), likely influenced by changes in kinetics and kinematic chaining as factors that have not been examined in the present investigation. However, the observed decrement in shooting accuracy disappeared 30 min post-completion of the upper-body resistance training session, implying on minor impact in a practical setting since resistance training sessions are not scheduled prior to the official game.

## 5. Conclusions

The results obtained in the present investigation indicate that performing upper-body and lower-body resistance training regimens prior to basketball on-court practice sessions, tailored toward maintaining an athlete's strength and power levels gained during the pre-season competitive period, has no impact on the 10 selected biomechanical parameters during free-throw, two-point, and three-point shooting motion and minor acute impact on mid-range and long-range shooting accuracy that lasts up to 30 min only post-upper-body exercise session completion. These findings may help coaches, sports scientists, and strength and conditioning practitioners with scheduling individual and team basketball and resistance training sessions targeted toward optimizing athletes' on-court basketball performance.

## Data Availability

The raw data supporting the conclusions of this article will be made available by the authors, without undue reservation.
